# A Recombinant Parathyroid Hormone‐Related Peptide Locally Applied in Osteoporotic Bone Defect

**DOI:** 10.1002/advs.202300516

**Published:** 2023-05-25

**Authors:** Yi Wang, Yingkun Hu, Shenghui Lan, Zhe Chen, Yufeng Zhang, Xiaodong Guo, Lin Cai, Jingfeng Li

**Affiliations:** ^1^ Zhongnan Hospital of Wuhan University Donghu Road 169 Wuhan 430071 P. R. China; ^2^ Department of Orthopaedics The Eighth People's Hospital Jiangsu University Shanghai 200235 P. R. China; ^3^ Department of Orthopaedics Xuhui Branch of The Sixth People's Hospital Shanghai Jiao Tong University Shanghai 200233 P. R. China; ^4^ Department of Orthopedics The Second Hospital of Tianjin Medical University Tianjin 300211 P. R. China; ^5^ Department of Orthopedics Union Hospital Tongji Medical College Huazhong University of Science and Technology Jiefang Road 1277 Wuhan 430022 P. R. China

**Keywords:** bioactive scaffold, bone remodeling, osteoblasts, osteoclasts, osteoporotic bone defect, parathyroid hormone‐related peptide

## Abstract

The local application of drug‐loaded bioactive scaffold materials is one of the important directions to solve the clinical problem of osteoporotic (OP) bone defects. This study retains the advantages of drug loading and mechanical properties of natural 3D bioactive scaffolds. The scaffolds are functionally modified through chemical and self‐assembly approaches with application of polydopamine (PDA) nanoparticles and parathyroid hormone‐related peptide‐1 (PTHrP‐1) for efficient local drug loading. This study investigates the effects of the novel bioactive scaffolds on ossification, osteoclastogenesis, and macrophage polarization. This work elucidates the effects of the scaffolds in regulating osteoclastic activity and new bone formation in vitro. Further studies on the establishment and repair of OP bone defects in small animals are conducted, and the potential of natural bioactive porous scaffold materials to promote the repair of OP bone defects is initially verified. The preparation of safe and economical anti‐OP bone repair material provides a theoretical basis for clinical translational applications.

## Graphical abstract

This paper develops a novel bioactive scaffold material by chemically and self‐assembled PDA nanoparticles co‐modifying CaP matrix scaffold with PTHrP‐1. This work verifies that PTHrP‐1 can be applied locally. For the first time, the positive facilitation effect of PTHrP‐1 local application is demonstrated during the repair of skull bone defects in osteoporotic rats, which may be attributed to the modification of recombinant PTH and the synergistic effect of PDA nanoparticles.

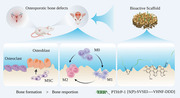

## 1 Introduction

Osteoporosis (OP) is a common metabolic bone disease caused by an imbalance in homeostasis within the bone tissue, which predisposes to adverse clinical outcomes such as increased fracture risk and fracture severity. Maintaining the structural stability of internal fixation in osteoporotic fractures is increasingly challenging.^[^ ^1^ ^]^ Bone defects are becoming more common in patients with osteoporosis, yet current treatments for bone defects in osteoporosis are unsatisfactory.^[^ ^2^ ^]^ The structural integrity of bone is severely compromised in patients with OP bone defects, further increasing the difficulty of bone defect repair because of poor bone quality, prolonged fracture healing, and high incidence of re‐fracture. Therefore, a key issue for such problems is how to effectively treat bone defects and improve bone microarchitecture. Researchers have attempted to apply various types of bone tissue engineering scaffolds, such as modified hydroxyapatite, *β*‐tricalcium phosphate, and calcium phosphate bone cement, all of which have been shown to promote bone regeneration.^[^ ^3^ ^]^ However, bone regeneration is severely impaired in the OP state, conventional scaffolds have limited ability to promote bone regeneration, the bone microenvironment of OP bone defects is complex (such as the occurrence of the inflammatory response and disordered bone metabolism), and the interaction with scaffolds affects the final effect of bone repair.^[^ ^4^ ^]^ Therefore, the construction of tissue‐engineered scaffold materials with multifunctionality for the problem of localized osteoporotic bone defects is a needed experimental exploration with clinical guidance.

Currently, the common treatment for osteoporotic bone defects is the combined use of anti‐osteoporotic drugs after orthopedic surgery. The surgical treatment options for patients with osteoporosis are mostly internal fixation and bone cement filling. Common complications in these patients include loosening of the internal fixation and secondary fractures of adjacent vertebrae. In addition, there is extracorporeal shock wave therapy, which stimulates microfracture at the fracture end by applying high pressure and rapid release of huge energy to induce the formation of microfine bone healing tissue, thus promoting bone repair.^[^ ^5^ ^]^ However, satisfactory results cannot be obtained in some patients with severe osteoporotic bone defects. The current conservative treatment for osteoporosis is mainly anti‐bone resorption and anabolic therapy.^[^ ^6^ ^]^ The main clinical drugs include bisphosphonates, calcitonin, estrogen, selective estrogen receptor modulators (SERMs), denosumab, and other drugs that inhibit bone resorption, as well as BMP‐2 and its derivatives, parathyroid hormone (PTH) and its derivatives, abaloparatide and other active factors that promote bone remodeling.^[^ ^7^ ^]^ Teriparatide, a derivative of the PTH, exhibits good anti‐OP biological activity but suffers from many disadvantages such as inadequate active site exposure, administration only by intermittent, systemic means, high cost, and tendency to induce hypercalcemia and osteosarcoma formation.^[^ ^8^ ^]^ Although great progress has been made in exploring the mechanism of action for reversing osteoporosis and improved treatment efficacy, many problems still exist in the repair of localized OP bone defects. The most critical issue in the development of anti‐osteoporosis drugs at this stage is the efficient loading of bioactive factors such that their good osteoinductive activity as well as their inhibitory osteoclastic activity is retained. In our team's previous modification and inflammation of PTH derivatives, we successfully developed a class of PTH‐related peptide drugs (PTHrP‐1/PTHrP‐2) which have been experimentally demonstrated to have good dual effects of osteoinductive activity promotion and osteoclastic activity reduction.^[^ ^9^ ^]^

**Figure 1:**
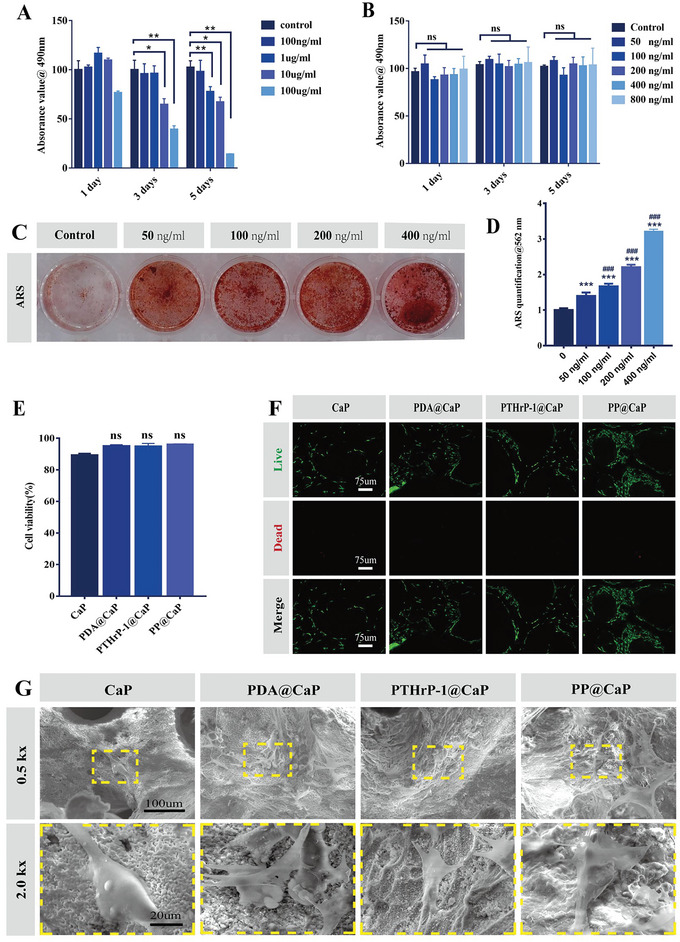
In vitro MC3T3‐E1 cytocompatibility of biofunctionalized scaffolds. A,B) Cell proliferation of MC3T3‐E1 cells at different concentration gradients on days 1, 3, and 5. C,D) Calcium crystallization of MC3T3‐E1 cells after 21 days of intervention with different concentrations of PTHrP‐1. E,F) Representative confocal images of live/dead staining and statistical analysis of MC3T3‐E1 cells. G) Representative SEM images of MC3T3‐E1 cells incubated with different scaffold materials for 3 days. Scale bars: 75 µm (F), 100 µm (G, low magnification SEM images), 20 µm (G, medium‐high magnification SEM images), *p < 0.05, **p < 0.01, and ***p < 0.001 indicate significant differences compared with the blank control group. # *p* < 0.05, # #*p* < 0.01, and ###*p* < 0.001 indicate significant differences compared with the high concentration group. Data are expressed as mean ± SD (*n* = 3).

With the iteration of high technology in chemical synthesis, the development of biofunctionalized composite scaffolds has become possible. Zhang et al. developed a layered bionic scaffold to modulate bone immunity and bone metabolism to promote bone regeneration by integrating polypropylene cross‐ester/hydroxyapatite, gelatin methacrylate‐based hydrogels, manganese‐carbon nanosheets and polycaprolactone nanoparticles.^[^ ^10^ ^]^ This scaffold mimics the structural features of gradients in cancellous and cortical bone tissue and has significant bone immunomodulatory properties.^[^ ^11^ ^]^ Zhu et al. developed a biofunctionalized composite scaffold using PLGE and nHA and coated with VEGF, which enhances osteoconduction, angiogenesis and beneficial metabolic microenvironment for the treatment of osteonecrosis.^[^ ^12^ ^]^ Artificial bone made of PLGA composites substitutes are also developing at a rapid pace.^[^ ^13^ ^]^ Drawing on these techniques, we intend to load anti‐osteoporotic agents (such as bisphosphonates, BMP‐2 and PTH‐related peptides) onto tissue‐engineered scaffolds for the treatment of localized OP bone defect repair. Dopamine(DA) and its derived composites are gradually being used in various fields because of their adhesive, reducing, and biocompatible properties which allow for common coating as well as secondary functionalized modifications. Polydopamine (PDA) can be coated on various substrates by the autoxidative polymerization of dopamine. PDA nanoparticles have favorable biocompatibility, adhesion properties and mechanical properties, moreover, they show excellent performance in free radical scavenging and UV shielding.^[^ ^14^ ^]^ PDA can modify various types of solid substrate materials and nanomaterials, and the surfaces of PDA‐coated scaffold materials have high adsorption capacities. In addition, our previous study^[^ ^9a^ ^]^ confirmed that increasing the concentration of PTH‐related peptides could promote osteogenic differentiation of mesenchymal stem cells (MSCs) and decrease osteoclast activity. With further study of drug concentration, this work confirmed that a higher concentration of PTH‐related peptide is not better but can promote osteogenic differentiation of MSCs more effectively. However, the cell proliferation activity gradually decreases with the increase of PTH‐related concentration when the maximum concentration of the drug that can be carried in the cell culture is exceeded (**Figure** **1**A,B). Surprisingly, osteogenic activity gradually increased with increasing concentrations of PTH‐related peptides, and the pro‐osteoclastic activity also increased rapidly; further, the bone resorption was greater than the rate of new bone formation, showing an overall net resorption phenomenon. Therefore, bioactive scaffolds with appropriate initial drug loading concentrations are essential for the repair of localized osteoporotic bone defects and the osseointegration of the material‐bone interface.

In our research, the advantages of self‐polymerized, highly viscous PDA and natural calcium‐rich ion bioactive scaffolds and the mechanical properties of natural porous scaffold materials were used to innovatively modify the surface and pore walls of bioactive scaffolds with PDA coating to achieve efficient local drug delivery. Therefore, investigating and developing new bioactive scaffold materials that meet clinical needs is necessary. The research and development of bioactive bone tissue engineering scaffold materials to meet clinical needs for mediating the adhesion and differentiation of relevant cells, improving the microenvironment of OP bone injury, and facilitating the regeneration and reconstruction of bone tissue are effective means to treat OP bone defects.

## 2 Results and Discussion

An increasing number of studies have shown that physical and chemical methods such as physisorption and covalent binding are often used to anchor bioactive molecules to bone implants and pore wall surfaces to induce new bone production and interfacial integration, thereby promoting bone regeneration and osseointegration.^[^ ^9^, ^15^ ^]^ However, physical adsorption may lose its effectiveness at long‐term implantation environments, mainly due to the low stability of the adsorbed molecules and limited adsorption efficiency. Covalent binding then frequently leads to reduced molecular bioactivity.^[^ ^16^ ^]^ The above surface modification methods for scaffold materials have mainly focused on the investigation of osteoblastic activity, while the effect on osteoclast activity has rarely been considered. Osteoclasts also play an important role in bone repair and bone remodeling of OP bone defects. Therefore, the lack of an ideal drug delivery system and limited osteoblast‐osteoclast differentiation activity will greatly limit its translation and application in patients with clinical OP bone defects. For this purpose, we describe a concise and efficient method for the preparation of organic‐inorganic coatings. The coating contains a stable sandwich – like hybrid structure of PDA nanoparticles and PTHrP‐1 co‐deposited on the CaP surface. Briefly, PDA nanoparticles were synthesized by mussel bio‐inspired dopamine chemistry and self‐assembly, and then co‐formed with PTHrP‐1 and CaP scaffolds to form sandwich – like hybrid multifunctional coatings. We designed and prepared a novel natural 3D bioactive scaffold with modified surface and pore walls. This novel bioactive scaffold has a synergistic effect on the treatment of osteoporotic bone defects by modulating osteoblast activity and osteoclast activity.

**Figure 2:**
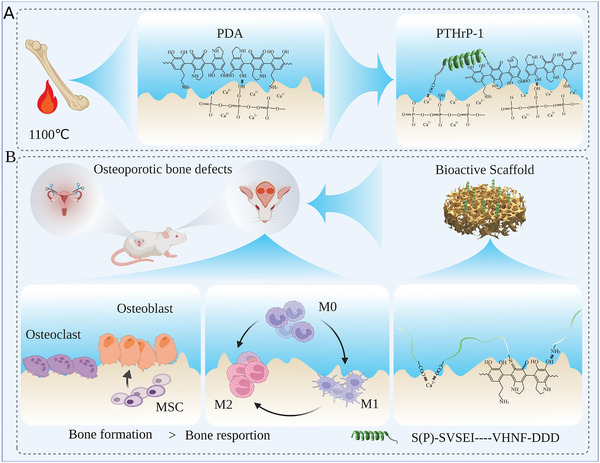
A) Schematic diagram of the preparation process of PP@CaP. For the preparation and evaluation of biofunctionalized calcium phosphate‐based materials for potential applications in osteoporotic bone regeneration. A novel surface‐modified 3D CaP matrix bioactive scaffold with a sandwich‐like hybrid surface loaded with PTHrP‐1 was prepared by bio‐inspired dopamine chemistry and self‐assembly method. B) Biofunctionalized PP@CaP scaffolds were evaluated in vivo and in vitro by locally applied PDA and PTHrP‐1 to promote endogenous bone regeneration in areas of osteoporotic bone defects through synergistic osteogenesis, inhibition of osteolysis, and M2 macrophage polarization. Created with BioRender.com

The adhesion of PDA on the material surface is mainly by two methods: covalent binding and non‐covalent binding. Among them, covalent binding is mostly based on the modified surface containing groups such as sulfhydryl (‐SH), amino (‐NH_2_), and carbonyl (—C=O) groups. The PDA is immobilized on the surface of the modified material by Michael addition or Schiff's base reaction under alkaline conditions. Non‐covalent binding is mostly based on the coordination or chelation of PDA with metal ions on the surface of the scaffold, hydrogen bonding, *π*—*π* conjugation and other interactions.^[^ ^17^ ^]^ The abundant functional groups of PDA make it bind to a variety of drugs or polymers, such as antibiotics, anti‐cancer drugs, and bioactive factors through electrostatic adsorption, *π*—*π* conjugation, and hydrogen bonding. Subsequently, PTHrP‐1 was introduced to the surface of PDA‐coated Calcium phosphate matrix (CaP) scaffold (PTHrP1@PDA@CaP, abbreviated as PP@CaP) through the interaction of PTHrP‐1(‐NH2) with many functional groups of PDA. And PDA binds PTHrP‐1 by Schiff base reaction of the carbonyl group (—C=O) in PDA with the amino group (‐NH2) in PTHrP‐1..The triple repeat aspartate sequence in PTHrP‐1 and the calcium‐rich ion in the CaP scaffold have natural high‐affinity binding sites that together build a sustainable drug release system. The negatively charged PDA can interact with PTHrP‐1 through electronic interactions and hydrogen bonding and act as a good sustainable drug release device at the site of injury (**Figure** **2**).

**Figure 3:**
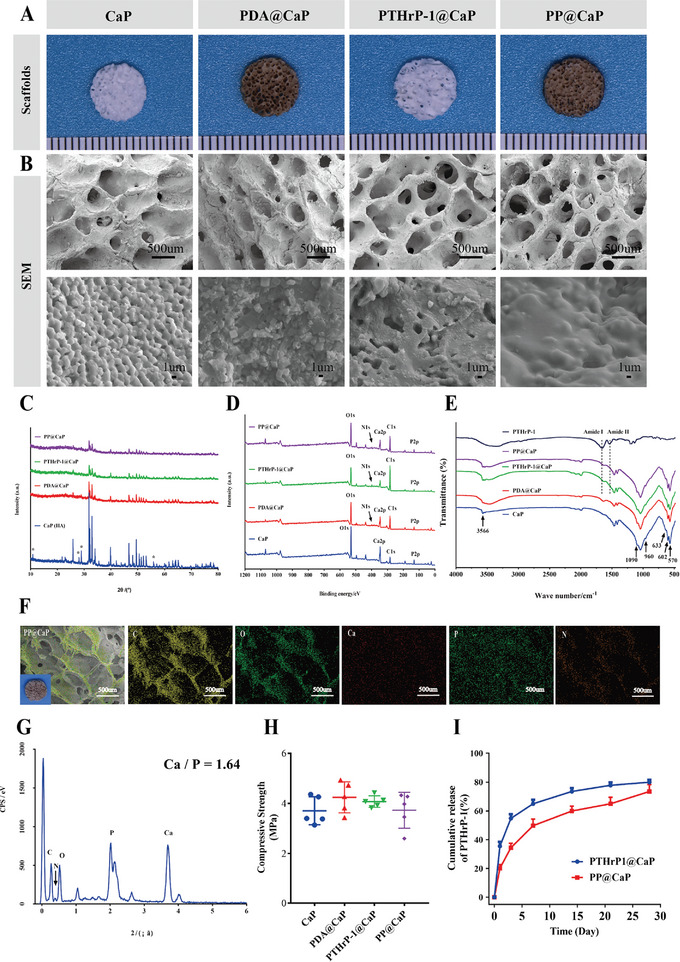
Characterization of different calcium phosphate‐based scaffold materials. A) Representative macroscopic photographs of different samples. B) SEM images of PP@CaP with porous interoperability and microstructure. C) X‐ray photoelectron spectroscopy (XRD) characterization was performed to analyze the material structure of different biofunctional scaffolds by diffraction effects in crystalline matter. D) X‐ray photoelectron spectroscopy (XPS) characterization was performed to confirm the surface of the pore structure of CaP scaffolds, and PTHrP‐1 was cross‐linked into PTHrP‐1@PDA scaffold by PDA. E) FTIR spectra of PP@CaP scaffold materials. F) EDX elemental mapping reveals the presence of Ca, P, C, O, and N elements. G) Ca/P ratio on the surface of PP@CaP scaffold. H) Statistical results of compressive strength at ≈70% strain. I) Release of PTHrP‐1 from PP@CaP scaffold. Scale bar: 500 µm (low‐magnification SEM images in B), 1 µm (high‐magnification SEM images in B), and 100 µm (EDS mapping images in F). Data are expressed as mean ± SD (*n* = 3).

In recent years, PDA coatings have been widely used for the surface functionalization of materials with various bioactive molecules (such as peptides, proteins, and related polymers) because of their mild synthesis conditions, high adhesion, biocompatibility, and easy immobilization of biomolecules.^[^ ^18^ ^]^ Given the adhesion properties of polyphenols, biopolymeric PDA coatings were formed on the surface of natural porous bioactive scaffolds by oxidative self‐polymerization under alkaline conditions. As shown in Figure 2, calcined calf bone derived CaP‐enriched bioactive scaffold was selected as the base part of the composite scaffold. It retains the natural interoperable porous structure and good mechanical properties, matches well with the defect cavity, and facilitates adequate coating of PDA and inward growth of new bone tissue. The PDA‐modified bioactive scaffold (PDA@CaP) was prepared by in situ self‐polymerization of dopamine using CaP and obtain a PDA biofunctional CaP scaffold (**Figure** **3**).

### 2.1 Preparation and Characterization of Biofunctionalized Scaffolds

CaP, intermediate PDA@CaP, PTH‐loaded PDA@CaP (PTH@PDA@CaP), PTHrP‐1 biofunctionalized CaP (PTHrP‐1@CaP), and PTHrP‐1 biofunctionalized P@CaP (PP@CaP) scaffolds were prepared for analysis. The appearance, surfaces, and microscopic morphologies of the scaffolds prepared by different processes were recorded by digital photographs and scanning electron microscopy (SEM) images (Figure 3A,B). As seen from the macroscopic photographs, the introduction of a PDA coating changed the surface color of CaP scaffolds from white to light brown, and no significant change was observed in the surface after modification by PTHrP‐1 coating. As reported in the literature, bone replacement materials should have proper porous 3D structure, porosity, and pore size to provide good space for adhesion, proliferation, and differentiation of various types of cells.^[^ ^19^ ^]^ Cross‐sectional SEM images at different magnifications showed the morphological characteristics of the scaffolds. All prepared scaffolds have an interoperable pore structure consisting of abundant macropores and micropores and retain the structural features of natural cancellous bone to some extent. These structures themselves may provide more adsorption sites for bioactive molecules, a feature which facilitates the transport of anti‐OP drugs, the improved transport of nutrients, growth of new bone, and rapid formation of implant interface integration. Through cell adhesion, proliferation, and tissue ingrowth, this 3D interoperable pore scaffold structure can induce the early osteogenesis of surrounding cells and tissues.^[^ ^20^ ^]^ High‐magnification SEM images also revealed uniformly smooth surfaces of CaP and PDA@CaP, and both PTHrP‐1@CaP and PP@CaP surfaces were uniformly wrapped by thin films of drug particles, indicating the presence of PTHrP‐1 crystals (Figure 3B). Given the porous interoperability and biofunctionalization of the surface, PTHrP‐1 can penetrate the inner surface of the bioactive scaffold instead of merely coating its outer surface. The sustained release of PTHrP‐1 facilitates the maintenance of the outcomes of effective drug concentrations on local cellular behavior as well as a lasting biological effect on the surrounding internal environment. The cumulative release profile of PTHrP‐1 was measured by HPLC‐MS, and the PP@CaP scaffold loaded with PTHrP‐1 maintained a sustained release over 28 days (Figure 3I). This stable sustained release trend may be related to the synergistic effect of PDA and triple repeat amino acid modifications. By contrast, the PTHrP‐1 release from the PDA@CaP scaffold used as a control reached 75% at day 7, probably arising from the rapid oxidation of the PTHrP‐1 coating and the consequent release of surface PTHrP‐1 molecules. These findings suggest that the prepared PP@CaP scaffolds can maintain a stable release of PTHrP‐1 molecules over a long period of time and that PTHrP‐1 molecules play a crucial role in the subsequent biological performance. The mechanical behaviors of the CaP, PDA@CaP, PP@CaP, and PP@CaP scaffolds were evaluated by compression tests. The CaP‐based bioactive scaffolds had similar compressive strength (Figure 3H) with no significant differences. We found no significant effect of the introduction of PDA and PTHrP‐1 coatings on the overall mechanical properties of the CaP scaffolds, a finding which may be caused by the low mechanical support provided by these components. In addition, the compressive strength of all scaffolds was ≈4 MPa, a feature that facilitates the promotion of cell proliferation and osteogenic differentiation.^[^ ^21^ ^]^ Given that the compressive strength of human cancellous bone ranges from 0.22 to 10.44 MPa,^[^ ^22^ ^]^ this biofunctional scaffold can be used to repair non‐bone bearing defect sites, such as the case for skull defect bone regeneration.

To determine the reaction between the PTHrP‐1, PDA, and CaP scaffolds, the samples were analyzed by EDS, FTIR, XPS, and XRD. The EDS assay results showed that the presence of elemental N was newly detected on the surface of the PDA‐modified as well as PTHrP ‐1 loaded bioactive scaffolds (Figure 3F,G). FTIR spectroscopy confirmed that the main characteristic absorption bands appeared at 1664, 1537, and 1090 cm^−1^, corresponding to amide I, amide II, and HA, respectively (Figure 3E). The absorbance of the primary amine group at 1570 cm^−1^ decreased significantly after PTHrP‐1 treatment, possibly because of the depletion of the primary amine group during the reaction. By contrast, the amide peak near 1640 cm^−1^ increased, indicating that the ester group on PTHrP‐1 reacted with the amino group on PDA to form a secondary amide. The 960–1090 cm^−1^ band reflects the antisymmetric stretching vibration of the PO4^3−^ group, and 570 and 633 cm^−1^ are counterparts for the PO4^3−^ group.^[^ ^23^ ^]^ Moreover, XPS detection (Figure 3D) revealed characteristic diffraction peaks of HA in all samples. Point to point XPS spectra are shown in Figure S1, Supporting Information). The crystalline phase of the PDA@PTHrP‐1 coating is consistent with the single phase of hydroxyapatite (HA, JCPDS No. 09–0169). The PP @ CaP coating has a crystalline peak similar to that of HA, which indicates that the modification of PDA and PTHrP‐1 did not reduce the original single‐crystal properties (Figure 3D). In addition, PTHrP‐1 would bind more stably on the surface of CaP scaffold materials with strong adsorption PDA and calcium‐rich ions.

### 2.2 In Vitro Cytocompatibility Evaluation of Bioactive Scaffolds

Previous studies have confirmed that appropriate concentrations of PTHrP can promote^[^ ^9b^ ^]^ cell proliferation and differentiation. The optimal PTHrP‐1 loading concentration was determined by in vitro osteogenic differentiation and cytotoxicity assays in MC3T3‐E1 cells. As shown in Figure 1C,D, the stronger osteogenic induction of PTHrP‐1 on MC3T3‐E1 cells was observed when the concentration was 0.4 µg mL^−1^, and no significant cytotoxicity was observed. On this basis, PTHrP‐1 at initial drug loading concentration of 0.4 µg mL^−1^ was selected to construct a PTHrP‐1‐loaded biofunctional scaffold. The present proliferation and adhesion experiments on MC3T3‐E1 and RAW264.7 cells revealed that the synergistic effect of MC3T3‐E1 differentiation and macrophage polarization by PP@CaP was achieved. The PDA‐coated modified CaP scaffold significantly increased the effective drug loading capacity of the scaffold and avoided the negative effect of high local PTHrP‐1 concentration on the bioactivity. Given its rich amino and amide groups, PDA not only provided new binding sites for PTHrP‐1 (which enabled PTHrP‐1 to be more immobilized on CaP) but also enabled CaP to have better bioactivity for M2 macrophage polarization at the initial stage of implantation.

To achieve effective bone regeneration repair, bioactive scaffolds for bone regeneration should be non‐toxic or minimally toxic and exhibit good cytocompatibility, which is a fundamental prerequisite for bone regeneration.^[^ ^24^ ^]^ This study employed as model cells the MC3T3‐E1 cells, which have been widely used in experimental studies for cytotoxicity evaluation, osteoblast differentiation, in vitro mineralization, and osteoblast mechanisms because of their stable and reproducible properties.^[^ ^25^ ^]^ To evaluate the in vitro cytocompatibility of these scaffolds, MC3T3‐E1 cells were inoculated onto these scaffolds and co‐cultured for 3 days before live/dead staining was performed. Live/dead staining results showed numerous live cells enriched within the walls of the scaffold pores (Cell viability >95%, Figure 1E,F), indicating good cytocompatibility of all scaffolds and a positive correlation between cell number and fluorescence intensity, further confirming that the PP@CaP group had many live cells, as indicated by the green color on day 3. This finding was mainly attributed to the porous 3D bioactive‐based scaffolds that provided sufficient space for cell growth. Furthermore, SEM images indicated that cells cultured on PDA@CaP, PTHrP‐1@CaP, and PP@CaP bioactive factor‐modified scaffolds tended to have more cellular pseudopod extensions, and the interaction of MC3T3‐E1 cells with these scaffolds confirmed that the modified scaffolds were not only non‐cytotoxic but also promoted 3D cell growth. This outcome mainly arises from the superior surface bioactivity, high porosity, good connected pore network structure, and surface biofunctionalization properties of PP@CaP. Then, we established a 3D cell culture model and examined the effects of different bioactive scaffold materials on the osteogenic differentiation ability of MC3T3‐E1. Alizarin red staining and Vonkossa results showed the strongest staining ability in the PP@CaP group, suggesting a superior osteogenic differentiation ability over the CaP material. The detection of osteogenic‐related genes also established that the expression of osteogenic‐related genes was higher in the PP@CaP group than in the CaP group.

**Figure 4:**
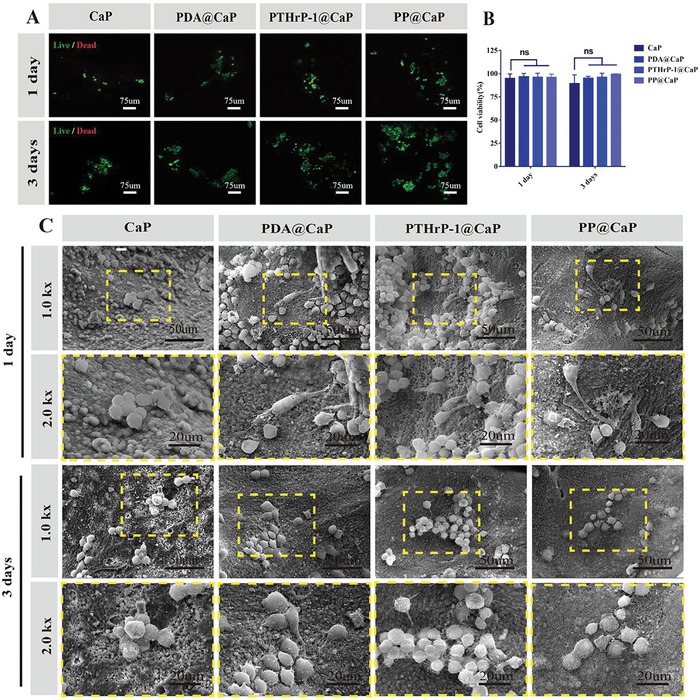
Compatibility of biofunctionalized scaffolds for in vitro RAW264.7 cells. A,B) Representative confocal images of live/dead staining and statistical analysis of RAW264.7 cells cultured in 3D under different biofunctionalized scaffold materials. C) Representative SEM images of RAW264.7 cells incubated for 1 day with different scaffold materials. Scale bars: 75 µm (A), 100 µm (C low magnification SEM image), 50 µm (C, medium‐high magnification SEM image), 20 µm (C, high magnification SEM image). Data are expressed as mean ± SD (*n* = 3).

Through live‐dead cell staining experiments, good biocompatibility was found in CaP, PDA@CaP, PTHrP‐1@CaP, and PP@CaP. MC3T3‐E1 and RAW264.7 cells had similar cell viability on the surfaces of PDA@CaP, PTHrP‐1@CaP, and PP@CaP, with PP@CaP having a better proliferation capacity (Figures 1E,F, and **4**A,B). This study also assessed the cell attachment and spreading morphology on the bioactive scaffolds by SEM (Figures 1G, and 4C). The observed SEM images reveal that all scaffolds were able to accommodate cell adhesion and proliferation, a finding which was attributed to a good microenvironment for cell growth because of the natural 3D porous interoperable structure. The cells cultured on the scaffolds after modification with PDA and PTHrP‐1 had good morphology with filamentous pseudopod extension and further firmly adhered to the scaffold surface. Notably, after 1 day of 3D co‐culture, the cells cultured on CaP scaffolds proliferated rapidly and were round or oval (Figure 4C), but the surfaces of PDA@CaP, PTHrP‐1@CaP, and PP@CaP showed shuttle and spindle shapes, which were the M1 phenotype. After 3 days of 3D co‐culture, more macrophages were present on the surfaces of PDA@CaP, PTHrP‐1@CaP, and PP@CaP, exhibiting a fried egg‐like shape and characterized as the M2 type. This outcome indicates that the cells form a strong interaction with the surface microstructure, consequently causing macrophages to polarize toward M2. For further validation, we also established a conditioned medium model and examined the effect on macrophage polarization of such a medium as induced by different bioactive scaffold materials. The surface macrophage M2 phenotype marker CD206 was higher in the PP@CaP group than in the CaP group per laser confocal immunofluorescence, and the M1 phenotype marker INOS was lower than CaP. Using RT‐PCR, the M1‐type macrophage marker INOS was expressed lower in PP@CaP, and the M2‐type marker CD206 was expressed higher therein. The PDA had some ability to promote macrophage polarization toward the M2 type, and the immune microenvironment generated by PP@CaP‐induced macrophages is conducive to enhancing the osteogenic differentiation of bone marrow MSCs. PDA has some ability to promote the polarization of macrophages toward the M2 type. First, although PDA has no amide groups, it contains many other bioactive functional groups, such as —C—OH, —N—H2, and C=O, which are involved in linking a variety of biomolecules, thus promoting the biological activity of M2.^[^ ^26^ ^]^ In addition, transcriptomic data reveal possible reasons why PDA promotes the polarization of M2 macrophages. PDA can upregulate the expression of anti‐inflammatory genes, such as Sfrp5, Wnt11, Fgf 16, Rab7b, Rab 15, ATF3, and Cd9, which contribute to the differentiation of M1 to M2 phenotype and the release of anti‐inflammatory cytokines.^[^ ^27^ ^]^ In addition, PDA has antioxidant capacity that scavenges ROS from damaged bone and inhibits the secretion of the pro‐inflammatory factor iNOS by M1 macrophages, thus favoring the polarization of M2.^[^ ^28^ ^]^ PP@CaP‐induced immune microenvironment generated by macrophages facilitates enhanced osteogenic differentiation of bone marrow MSCs.

In summary, PP@CaP can show great potential for orthopedic implants by providing a highly biocompatible microenvironment for the adhesion, proliferation, and differentiation of MC3T3‐E1 and RAW264.7 cells through its biologically functional surface properties.

### 2.3 In Vitro Osteogenic Evaluation

The ideal bone repair implant should not only have good cytocompatibility but also exhibit excellent osteogenic activity. Encouraged by the above 2D and 3D cell‐scaffold co‐culture results, we evaluated the adhesion and proliferation ability of the MC3T3‐E1 cells on these biofunctionalized scaffolds.

**Figure 5:**
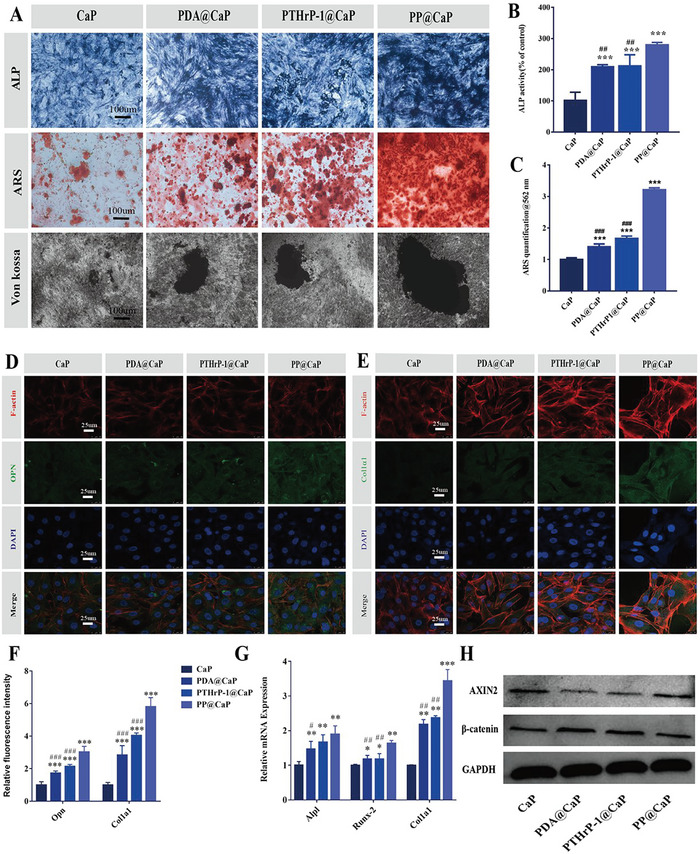
Study of in vitro osteogenic activity of biofunctional scaffolds. A) Representative images of ALP staining, ARS staining and Von Kossa staining of different scaffold samples 7, 21, and 28 days after cell seeding. B,C) Quantitative evaluation of ALP activity and matrix mineralization in MC3T3‐E1 cells on different scaffold samples. D–F) Representative immunofluorescence staining images of osteogenic OPN and Col‐1*α*1 proteins after 7 days of culture. Target proteins, f ‐actin and nuclei were labeled with fluorescent green, red, and blue, respectively, as well as quantitative analysis. G) Relative mRNA expression levels of the osteogenesis‐related markers ALP, Col‐1*α*1 and Runx2 after 7 days of culture. H) Representative western blot results of *β*‐catenin and AXIN2 in MC3T3‐E1 cells co‐cultured with various scaffolds or scaffold extracts. Scale bars: 100 µm (A), 25 µm (G–E, medium‐high magnification CLMS images). *p < 0.05, **p < 0.01, and ***p < 0.001 indicate significant differences compared with the blank control group. ^#^ *p* < 0.05, ^##^ *p* < 0.01, and ^###^ *p* < 0.001 indicate significant differences compared with the high concentration group. Data are expressed as mean ± SD (*n* = 3).

To explore the effect of biofunctional scaffolds on ossification, we evaluated its effect on osteogenic differentiation of MC3T3‐E1 cells by detecting ARS, Von Kossa, and the expression of osteogenic‐related genes. Calcium deposition is an important indicator of osteogenic ability. To directly observe calcium deposition, MC3T3‐E1 cells were stained with ALP, ARS, and von Kossa after 7, 14, and 28 days of co‐culture, respectively. According to the ALP, ARS, and von Kossa results, the PP@CaP scaffold showed more significant calcium deposition and expressed more mineralized matrix than the CaP, PDA@CaP, and PTHrP‐1@CaP scaffolds, suggesting that PP@CaP had osteogenic advantages (**Figure** **5**A–C), These results indicated that the efficient loading of PTHrP‐1 was more favorable to induce osteogenic differentiation of MC3T3‐E1 cells. Moreover, the ALP activity and calcium deposition of MC3T3‐E1 cells were highest in the PP@CaP scaffold, followed by the PTHrP‐1@CaP scaffold, in accordance with our previous hypothesis (Figure 5B,C). Although ALP activity was slightly elevated in the PDA@CaP group compared to the CaP group, the difference was not significant.

In addition, Col‐1 and OPN were detected by immunofluorescence to detect the expression of osteoblast proteins in MC3T3‐E1 cells. As shown in Figure 5D,F, OPN protein (green) is abundant in the PP@CaP group. In contrast, the expression of OPN protein was lower in the CaP and PDA@CaP groups. Furthermore, Col‐1 immunofluorescence staining showed a similar trend, with the highest protein content in the PP@CaP group, followed by the PTHrP‐1@CaP group (Figure 5E,F), few amounts of OPN protein were also observed in the CaP and PDA@CaP groups at similar levels.

To further verify the osteogenic ability of scaffolds, qRT‐PCR was used to detect the expression of osteogenic genes after co‐culture with different scaffolds. The results showed that PP@CaP up‐regulated the expression of osteogenic genes in MC3T3‐E1 cells, which would be beneficial to the repair of bone defects (Figure 5G). It is well known that the physiological process of osteogenesis involves a large number of regulatory factors, such as Runx2, Col‐1, and Alpl. Runx2 is a member of the transcription factor runt homologous domain family and plays a key role in the regulation of osteoblast gene expression. Col‐1 is a member of Group I collagen and plays an important role in collagen deposition. In addition, we demonstrated that PP@CaP scaffolds effectively activate the Wnt/*β*‐catenin signaling pathway (Figure 5H), an important pathway for osteogenic differentiation, suggesting that sustained PTHrP release ultimately enhances the osteogenic effect. Studies have shown that both estrogen and PTH promote the expression of the Wnt/*β*‐catenin pathway, but the regulatory mechanisms are unclear. There are two possible mechanisms for the increased expression of Wnt/*β*‐catenin. The first mechanism is that PTH exerts a direct regulatory effect on cells through PTH receptors, regulating the expression of Axin2 and GSK‐3*β*, key proteins of the Wnt/*β*‐catenin pathway, or reducing the expression of DKK‐1 or SOST, Wnt/*β*‐catenin inhibitors, to promote Wnt/*β*‐catenin signaling. Another mechanism is that the Wnt/*β*‐catenin signaling pathway is highly expressed in osteoblasts in proportion to the number of osteoblasts, and PP@CaP scaffolds increase the number of osteoblasts while enhancing the expression of Wnt.^[^ ^29^ ^]^ These results further suggest that PP@CaP scaffolds with a PDA coating and which can sustainably release PTHrP‐1 molecules are more effective in inducing differentiation of MC3T3‐E1 cells toward osteogenesis.

### 2.4 In Vitro Evaluation of Osteoclast Formation

Like osteoblasts, osteoclasts play an indispensable role in the bone healing process.^[^ ^30^ ^]^ The role of osteoclastic resorption in OP bone defects is often greater than in the bone formation process, and reversing or reducing osteoclastogenesis may have a positive effect against osteoporosis and bone regeneration. Therefore, evaluation of the anti‐osteoporosis and bone regeneration ability of bioactive scaffold materials involves not only their osteogenic differentiation ability but also their effect on osteoclast differentiation. Given that mouse bone marrow macrophages (BMMs) are the main and important cell source for osteoclast differentiation,^[^ ^4^ ^]^ they were chosen as model cells in this study to evaluate the effect of these bioactive scaffolds on osteoclast activity. After isolation of the extracted BMMs, the cells were identified using flow cytometry and had a high percentage of CD11b (≈87%), which matched the characteristic markers of macrophages (Figure S2, Supporting Information).

**Figure 6:**
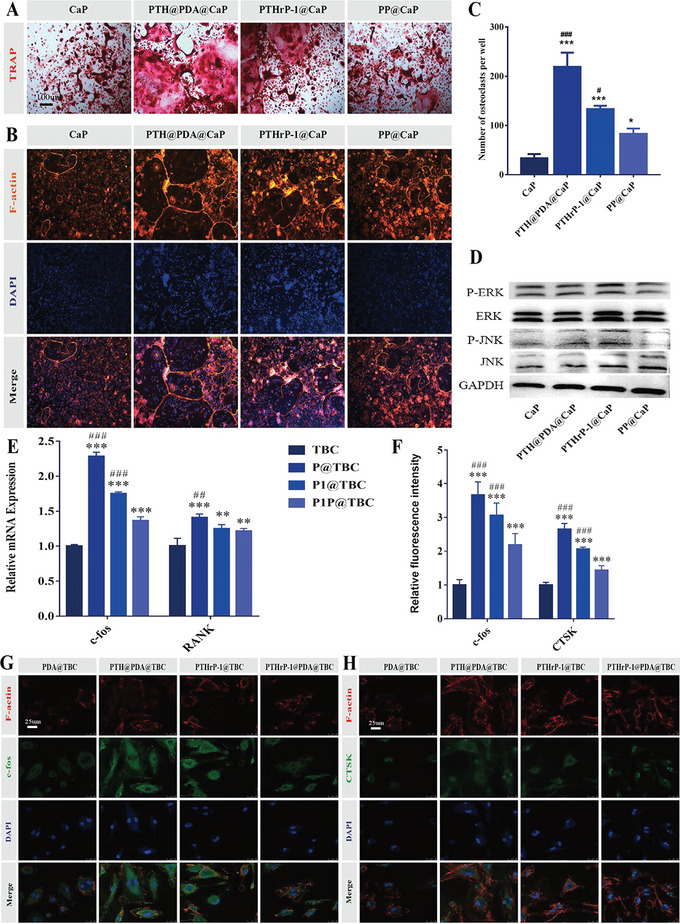
Evaluation of the anti‐osteoclast activity of different biofunctional scaffolds in vitro. A,C) Representative TRAP staining images and quantitative analysis results. B) Representative images of f ‐actin ring formation assay. E) Relative mRNA expression levels of osteoclast‐related markers c‐fos and CTSK after 3 days of culture. D) Representative western blot results of JNK and ERK in BMMs co‐cultured with various scaffolds. Quantitative analysis of the corresponding protein levels obtained by western blot. F–H) Representative immunofluorescence staining images of osteoclast c‐fos and CTSK proteins after 3 days of culture. Target proteins, f ‐actin and nuclei are labeled with fluorescent green, red and blue, respectively. Scale bars: 100 µm (A), 250 µm (B), 25 µm (G,H). Data are expressed as mean ± SD (*n* = 3). *p < 0.05, **p < 0.01, and***p < 0.001 indicate significant differences compared with the CaP group. ^#^ *p* < 0.05, ^##^ *p* < 0.01, and ^###^ *p* < 0.001 indicate significant differences compared with the PP@CaP group.

To explore the effect of biofunctional scaffolds on osteoclastogenesis, we detected its effect on osteoclast differentiation by TRAP, phalloidin staining and the expression of osteoclast – related genes. The results showed that the PP@CaP group showed a significantly reduced area of TRAP‐positive cells compared to PTH@PDA@CaP and PTHrP‐1@CaP and exhibited lower osteoclast differentiation (**Figure** **6**A,C). It is well known that the f‐actin ring serves as a dynamic and characteristic cytoskeletal structure of active osteoclasts, as subsequently verified by f‐actin ring immunofluorescence experiments. As shown in Figure 6B, multinucleated cells with well‐defined f‐actin rings were observed in the PTH@PDA@CaP group, but f‐actin rings were limited and restricted in the CaP, PTHrP‐1@CaP, and PP@CaP groups. Note that the area of f‐actin ring formation was significantly reduced in the PP@CaP group, an outcome which may be mainly attributed to the avoidance of the short burst release of PTHrP‐1 in the biofunctional scaffold. This finding was confirmed by the comparative results of other experimental groups, and the efficient piggybacking and slow‐release properties of PTHrP‐1 may be the most important reason for the reduced osteoclast activity of the composite bioactive scaffold.

Moreover, the phosphorylation levels of JNK and ERK were decreased in PP@CaP group (Figure 6D), which indicated that PP@CaP could inhibit the activation of MAPK signaling pathway and thus affect osteoclast differentiation. Indeed, As shown in Figure 6E, qRT‐PCR showed that osteoclast‐related genes in the PP@CaP group were significantly down‐regulated during culture compared with those in the PTH@PDA@CaP and PTHrP‐1@CaP groups. The expressions of c‐Fos and CTSK proteins were further observed by immunofluorescence (Figure 6F–H). The results also showed that the expressions of c‐Fos and CTSK in the PP@CaP group were lower than those in the PTH@PDA@CaP and PTHrP‐1@CaP groups. In addition, the fluorescence intensity of c‐Fos and CTSK staining was weak, indicating that osteoclast activity was inhibited, which was consistent with the results of qRT‐PCR detection. Together, these results suggest that PP@CaP scaffolds significantly inhibit osteoclast formation.

### 2.5 In Vivo Evaluation of Bone Regeneration

**Figure 7:**
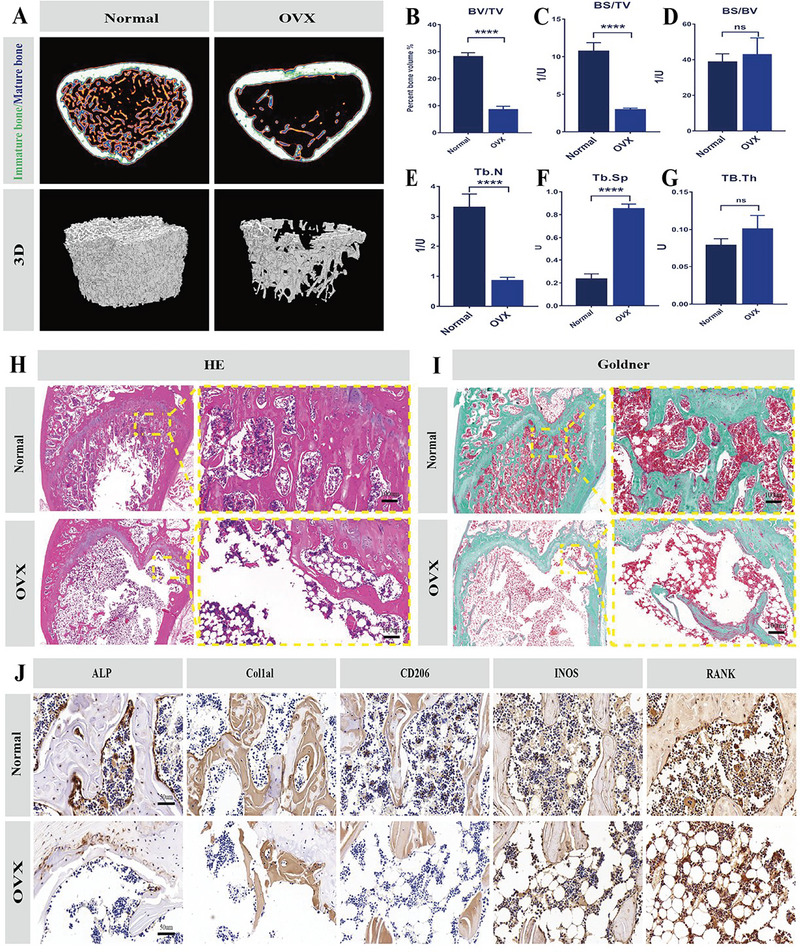
Modeling and validation of osteoporosis. A) Micro‐CT scan was performed on the femur sample of the rat, which confirmed the success of the osteoporosis model of the rat after ovary removal. B–G) Quantitative statistical analysis from bone trabeculae, bone cortex, and bone volume by BV/TV, BS/TV, BS/BV, Tb.N, Tb.Sp, TB.Th, etc. H) HE staining; I) tartaric acid staining; J) immunohistochemical staining, target genes were Alpl, CoL1*α*1, RANK, INOS, CD206. Scale bars: 1 mm (A), 100 µm (H–I), 50 µm (J). Data are expressed as mean ± SD (*n* = 3). *p < 0.05, **p < 0.01, and ***p < 0.001 indicate significant differences compared with the VOX group.

Given that PP@CaP has good cytocompatibility, has the ability to contribute to bone formation and reduce osteoclastogenesis, and provides a good immune microenvironment, we further explored it's in situ bone repair ability. To determine the bone regeneration ability of the novel bioactive scaffold in the in vivo OP bone defect environment, the bilateral ovaries of rats were first removed to construct an osteoporosis model. 12 weeks after operation, the bone cortex and trabeculae and the condition of the distal femur were assessed by imaging and histological analysis. Micro‐CT images showed smaller bone trabeculae in the ovariectomized (OVX) group compared to the sham‐operated control group 12 weeks after ovary removal (**Figure** **7**A). Further quantitative data analysis using BV/TV, BS/TV, BS/BV, Tb.N, Tb.Sp, and Tb.Th showed that the OVX group had fewer bone trabeculae, bone cortex, and bone volume, confirming the successful construction of the osteoporosis model (Figure 7B–G). The novel bioactive scaffolds were then implanted in situ into a critical size, 5 mm diameter cranial defect in rats. Histological stainings, such as HE staining and Goldner staining, further confirmed that the OVX group had reduced bone trabeculae, disturbed bone structure, and increased fatty and fibrous tissue(Figure 7H,I). To further explore the internal microenvironment of osteoporosis, immunohistochemical analysis of ALP, Col1*α*l, RANK, INOS, and CD206 was performed 12 weeks after surgery (Figure 7J).

**Figure 8:**
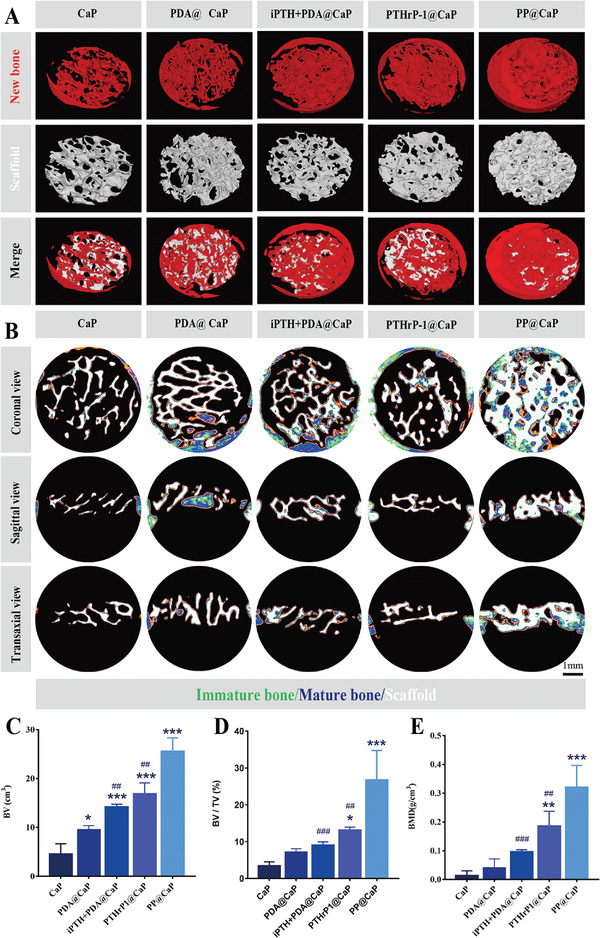
Radiographic analysis of bone formation in vivo. A) Representative 3D microscopic CT image of the skull defect 12 weeks after implantation. The yellow dashed line indicates the border of the defect (5 mm in diameter). B) Distribution of new bone tissue in each group at 12 weeks post‐implantation. C–E) Quantitative analysis of microscopic ct results of new bone tissue in the bone defect area, including BV, BV/TV, and BMD. Scale bars: 1 mm (A,B). Data are expressed as mean ± SD (*n* = 3). *p < 0.05, **p < 0.01, and ***p < 0.001 indicate significant differences compared with the CaP group. ^#^ *p* < 0.05, ^##^ *p* < 0.01, and ^###^ *p* < 0.001 indicate significant differences compared with the PP@CaP group.

The new bioactive scaffold was then implanted in situ into a 5 mm diameter cranial defect of critical size in rats (**Figure** **8**). Micro‐CT images can vividly and precisely show the bone repair effect. As shown in Figure 8A, the 3D reconstructed Micro‐CT images showed that all bone defects implanted with CaP‐based scaffolds had different degrees of regenerative new bone formation after 12 weeks. Consistent with our expected results, the PTHrP‐1 functionalized scaffold group had more new bone growth than the other groups, indicating a positive effect of PTHrP‐1 on bone healing. Note that PP@CaP implantation achieved the best bone defect repair results and revealed significant material bone interface integration and bone defect bridging at 12 weeks. These results suggest that PDA and PTHrP‐1 have a synergistic effect in accelerating new bone formation. In addition, 2D reconstructed images demonstrated more pronounced new bone ingrowth and material bone interface integration in the PP@CaP group than in the PTHrP‐1@CaP and other groups, with substantial new bone tissue growth between the PP@CaP scaffold material and the bone interface, a growth which penetrated the scaffold surface and its interior (Figure 8B). 2D micro‐CT results in the coronal plane confirmed that some areas of newly formed bone tissue were tightly connected to the bone interface and spread to the interior of the scaffold along the 3D interpenetrating pores. Moreover, transaxial 2D CT imaging of the defect area established that the new bone tissue in the PP@CaP group had fully grown between the defect and the bone interface. The above findings suggest that the PP@CaP scaffold can provide a beneficial microenvironment for osteoblasts through the synergistic effect of PDA and PTHrP‐1, thus effectively completing bone regeneration. Further, PTHrP‐1 regulates bone formation by activating the Wnt/*β*‐catenin signaling pathway, which plays an important role in regulating osteoblast‐mediated bone formation and osteoclast‐mediated associated bone resorption. We also found that CaP and PDA@CaP scaffolds were less effective for bone regeneration and osseointegration. Therefore, in the absence of osteogenic induction, CaP and PDA@CaP scaffolds would serve to provide space for osteogenesis without sufficient osteogenic induction, but this role is limited in OP bone defect model in SD rats. To further analyze and confirm the above findings, bone morphological parameters (BV, BV/TV, and BMD) were quantified (Figure 8C–E). Using the iPTH+PDA@CaP and PP@CaP scaffold groups showed significantly higher levels of BV, BV/TV, and BMD than the other three groups, with the highest values in the PP@CaP group. Thus, the PP@CaP scaffold had the fastest and best osteogenic bioactivity while minimizing the reduction of osteoclastogenic activity; these results indicate conduciveness toward favorable bone formation. The above findings suggest that the PP@CaP scaffold with the introduction of PTHrP‐1 and PDA plays a positive synergistic role in promoting bone formation.

**Figure 9:**
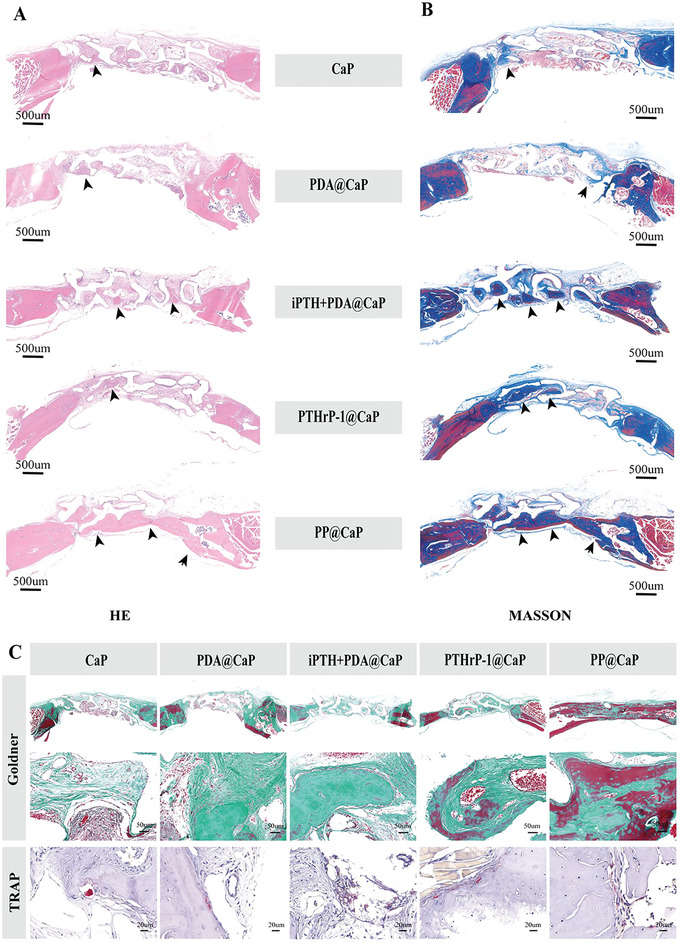
In vivo immune response. A,B) H&E staining and Masson staining images in the defective area 12 weeks after implantation. Black arrows indicate newly formed immature and mature bone tissue in the defect area. C) Representative Goldner staining and TRAP staining images of the defect site. Analysis of new bone formation, mineralized bone and bone resorption. scale bars: 500 µm (A,B), 50 µm (C, Goldner), 20 µm (C, TRAP).

Histological analysis was performed to assess the bone formation pattern at the defect site 12 weeks after implantation. The H&E staining results showed that only a thin layer of fibrous tissue was formed in the control group, with a small amount of calcified tissue developing in the peripheral area (**Figure** **9**A). This indicates that for the CaP and PDA@CaP groups, the bone defect areas were mostly covered with undegraded material and scattered local immature bone without forming an overall bone bridge, suggesting that the interconnected porous structure within the scaffold has a positive effect on bone regeneration. In contrast, in the iPTH+PDA@CaP, PTHrP‐1@CaP, and PP@CaP groups, extensive new bone tissue was visible around the pore channels, especially in the PP@CaP group, where the degree of bone mineralization was close to that of the surrounding native bone tissue. Notably, the center of the PP@CaP scaffold as well as the surrounding area was almost filled with newly formed bone tissue, which also indicated that the modification of PDA and the sustained release of PTHrP‐1 synergistically promoted osteogenic differentiation and mineralization within the scaffold, ultimately improving the osteoporotic bone defect repair. These data were consistent with micro‐CT results. Masson trichrome staining showed that the defect areas of the PDA@CaP and CaP groups were filled with a large number of collagen fibers, fibrous tissue, and scattered distribution of immature bone tissue (blue). On the other side, new bone (red) was significantly more mature in the PP@CaP group than in the PTHrP‐1@CaP group (Figure 9B). The PTHrP‐1@CaP group clearly had more blue areas indicating collagen fibers and immature bone. The observations verified that bone defect repair was enhanced and accelerated in both the PTHrP‐1@CaP and PP@CaP groups compared to the other groups, and that the PP@CaP group showed better osteoporotic bone repair characteristics compared to the other groups, in accordance with the expected results. In vivo bone mineralization and bone resorption were also further investigated by Goldner's trichrome staining and TRAP staining (Figure 9C). Not surprisingly, a large amount of dark green mineralized bone was observed in the defect area in the iPTH+PDA@CaP, PTHrP‐1@CaP, and PP@CaP groups, especially in the PP@CaP group, with fewer TRAP‐positive osteoclasts (stained purplish red), which confirmed that the PP@CaP scaffold promotes new bone maturation during bone repair while inhibiting bone resorption. In contrast, sparse immature bone (bone‐like, stained orange/red) and more osteoclasts were detected in all other groups at 12 weeks post‐implantation. These results suggest that bioactive scaffolds loaded with PTHrP‐1 may serve as good candidates for regulating osteoblast/osteoclast differentiation, thus exerting a synergistic anti‐osteoporosis and bone regeneration‐promoting effect.

This study utilizes the properties of inorganic calcium phosphate bone scaffolds to obtain biologically active biomaterials that meet the requirements of endogenous bone regeneration and enhanced implantation, such as for osseointegration strategies. In osteoporosis formation, osteoclasts interact with various osteoblasts and endothelial cells to promote the onset and aggravation of osteoporosis. Moreover, PTH‐dependent osteogenesis induction has a considerable impact on reversing osteoporosis. Our previous study confirmed that the modified PTH retains good osteogenic and angiogenic properties and has some side effects such as reducing the excessive osteoclast activity and pro‐inflammatory factor release induced by PTH itself, but the mechanism of action has not been further explored. In the present study, we further investigated its effect on osteoblastic activity and the regulation of pro‐inflammatory macrophage polarization based on our prior work and further examined the mechanism of its effect on reducing osteoclastic activity, with the aim of using PTH to tilt the balance of osteogenesis and osteoclastic metabolism in the direction of osteogenesis and consequently achieve the effect of osteogenesis promotion and osteolysis inhibition. Surprisingly, aside from the continuous local delivery of bioactive factors in small doses, the positive effect of reversing osteoporosis was successfully achieved. To explore the effectiveness of its modified nanoparticles, the PP@CaP modified CaP scaffold was subsequently applied in the treatment of OP bone defect repair. PP@CaP exerted a synergistic positive effect of reversing osteoporosis and osseointegration in the region of OP bone defects compared with the CaP scaffold alone, thereby realizing the local application of PTHrP in OP bone defect repair treatment and providing a new therapeutic idea for Calcium phosphate matrix drug‐loaded materials in the treatment of OP bone defects.

## 3 Conclusions

In summary, novel biofunctionalized PP@CaP 3D scaffolds with appropriate physicochemical properties were successfully fabricated by combining the strategies of bio‐inspired dopamine chemistry and self‐assembly, as well as employing triple repeat aspartate sequences with naturally high affinity for calcium ions to effectively combat osteoporosis and enhance bone regeneration and osseointegration in bone defect areas. The CaP scaffold acts as a macroscopic framework providing mechanical support and inorganic components, and the sandwich‐like hybrid surface loaded with PP@CaP confers bioactive properties such as cell adhesion, proliferation, and differentiation through the stabilization and long‐term sustained release of PP@CaP. In vitro cellular assays showed that PP@CaP has significant pro‐osteogenic and anti‐osteoclastic abilities. Crucially, in vivo OP bone defect repair and osseointegration assessments further reflected these results and confirmed that the transplanted biofunctionalized CaP scaffold induced favorable osteogenesis and inhibited osteoclastogenesis, ultimately reversing bone metabolism, effectively inhibiting further osteoporosis progression, and accelerating in situ osseointegration. Therefore, this multifunctional CaP implant is well‐suited for orthopedic applications. Finally, this work provides a green, convenient, and low‐cost strategy for scaffold surface modification, and the remarkable biological functions of multifunctional CaP implants have great potential for clinical translation.

## 4 Experimental Section

### Reagents and Materials

Dopamine hydrochloride (purity ≥98%) and tris(hydroxymethyl)amine methane (Tris) were purchased from Sigma‐Aldrich Trading Co Ltd (Shanghai, China). Mouse skull‐derived MC3T3‐E1 preosteoblasts were purchased from the Cell Bank of the Chinese Academy of Sciences (Shanghai, China). Fetal bovine serum (FBS), phosphate buffer (PBS), Trypsin‐EDTA, Penicillin/Streptomycin (P/S), *α*‐modified Eagle's medium (*α*‐MEM), and Dulbecco's modified Eagle's medium (DMEM) were purchased from Gibco Life Technologies Co.(Grand Island, USA). MTT Cell Proliferation and Cytotoxicity Assay Kit was purchased from Shanghai Aladdin Co.(Shanghai, China). Live/dead cell staining kits were purchased from BestBio Biotechnologies (Shanghai, China). The nuclei and cytoskeleton were stained with DAPI (Sigma‐Aldrich) and ghost pen cyclic peptide (Invitrogen). TRIzol RNA Extraction Kit and 5‐bromo‐4‐chloro‐3‐indolyl phosphate/nitro blue tetrazolium (BCIP/NBT) alkaline phosphatase (ALP) color development kit were purchased from Jiangsu Beotech Biotechnology Co. (Jiangsu, China).Von Kossa staining solution was purchased from Solarbio Co.(Beijing, China). Tartrate resistant acid phosphatase (TRAP) staining solution was purchased from Servicebio Co. (Servicebio, Cat# G1050). Autonomous synthesis of calcium‐phosphorus ceramic bone scaffold for calves.

### Hole Wall Modification of Porous Scaffolds

Natural CaP‐enriched bioactive scaffolds from fresh calf cancellous bone were sintered at 1200°C for 2 h in a microcontroller‐controlled Muffle furnace (Hefei Kocrystal Co., Anhui, China) at a temperature increase rate of 2 °C min^−1^, followed by natural cooling. After ultrasonic cleaning 2–3 times, 15 min per time, and then after hot air drying at 60 °C, reserve. With reference to the experience of previous studies, PTHrP‐1 was synthesized by the FMOC/tBu solid‐phase synthesis method. In this study, pyrophosphate, PDA and PTHrP‐1 were used to modify the pore wall surface of the scaffolds. The concentration and soaking time of pyrophosphate used for synthesis were obtained from a series of preliminary experiments and evaluated by XPS (Figure S1, Supporting Information)). Optimal reaction conditions were obtained by soaking in 10% solution at 60 °C for 60 min. In brief, the raw materials were dissolved in deionized water in a specific order at 10 min intervals for 60 min, thickened and uniformly coated on the surface of the scaffold, and modified by sintering for 3 h at 650 °C. Tris‐HCl buffer (10 mm, PH 8.5) was used to prepare 2.0 mg mL^−1^ PDA solution, and the bioactive scaffold was immersed in PDA solution and shaken for 24 h. After removal, they were rinsed three times with deionized water to remove unadsorbed dopamine. The fume hood was dried for 12 h, then the scaffolds were soaked in 50 ug mL^−1^ PTHrP‐1 solution for 12 h, centrifuged, and freeze‐dried. The material modification process was obtained in a sterile environment.

### Characterization of Scaffolds

Digital camera (80D, Canon, China) was used to take pictures of the appearance morphology of the scaffolds before and after modification. All scaffold samples were coated with gold on the surface and examined by field emission scanning electron microscopy (FE‐SEM, Zeiss, SIGMA, Germany) at an accelerating voltage of 10 kV, where the surface microstructural features of the samples included surface morphology, pore structure, and pore size. The samples were analyzed for surface elements using an X‐ray photoelectron spectrometer (XPS, ESCALAB 250XI, Thermo Scientific, USA) and an energy spectrometer (EDS, UltimMax 40, Oxford, UK). The chemical state of the samples (*λ* = 1.540598 Å) was analyzed by Fourier transform infrared spectroscopy (FTIR, TNZ1‐5700, Nicolet, USA) and X‐ray diffraction (XRD, D/max 2550 V, Rigaku, Japan). A versatile testing machine (CMT6503, Shenzhen, China) was used to test the compressive strength of the samples. To observe the in vitro release process of PTHrP‐1 from bioactive scaffolds, innovative synthesis of fluorescein isothiocyanate (FITC)‐labeled PTHrP‐1 was performed. Freeze‐dried scaffold samples were immersed in 10 mL PBS solution (pH = 7.4) at 37 ± 1 °C and shaken at 70 rpm. At different time points, the supernatant was removed for analysis and replaced with fresh PBS for detection and analysis by confocal laser confocal microscopy (CLSM; TCS SP8, Leica, Germany) with a 488 nm (green) excitation filter.

### Cell Culture

To test the cytocompatibility, MC3T3‐E1 and Raw264.7 cells (Cell Culture Center, CAS) were seeded on the surface of porous samples and cultured in scaffold extracts, respectively. Scaffold extracts were obtained from DMEM (Gibco BRL, USA) or ECM (Gibco BRL, USA) medium with scaffolds for 48 h and then filtered through 0.22 µm syringe filters. In addition, cell proliferation and cell morphology were determined by CCK‐8 and RT‐PCR analysis. For in vitro osteogenic potential testing, MC3T3‐E1 cells were treated with osteogenic inducible *α*MEM (50 mg mL^−1^ L‐ascorbate, 10 mm glycerol phosphate, and 100 nm dexamethasone). HuVEC cells were grown in scaffold extract (ECM) to promote cell proliferation. Medium containing 10% FBS (Gibco BRL; USA) and 100 U mL^−1^ penicillin‐streptomycin (Thermo Fisher Scientific, MA, USA). Cells were cultured on porous scaffolds (ø, 8 × 2 mm) in 24‐well plates at a density of 5 × 10^5^ per well. They were incubated at 37 °C for preset time stages (2 days for morphological analysis, 3 and 7 days for RT‐PCR analysis, 7 days for alkaline phosphatase (ALP) staining, and 21 days for alizarin Red S (ARS) staining).

### Screening of the Optimal PTHrP‐1 Initial Loading Volumes

To obtain the optimal PTHrP‐1 loading amount for constructing the bioactive scaffold surface, the optimal PTHrP‐1 initial loading concentration range (≈50 ng mL^−1^–100 ug mL^−1^) was used to screen the optimal PTHrP‐1 initial loading concentration for preparing PTHrP‐1 loaded scaffolds. Briefly, MC3T3‐E1 cells were first inoculated at a density of 1 × 10^4^ cells per well in a 96‐well plate containing 10% FBS, 1% P/S in *α*MEM complete medium, and then different initial concentrations of PTHrP‐1 were added. After 1, 3, and 5 days of incubation, the cells were incubated with 10 µL per well, MTT (5 mg mL^−1^) solution for 4 h in a light‐proof environment, that is, the MTT method was used to Cell viability was detected. The absorbance at 570 nm was measured using an enzyme marker (Multiskanfc, Thermo Scientific, USA). ARS staining was performed according to the manufacturer's instructions to assess osteogenic activity. To quantify calcium deposition, samples to be stained were treated with 10% ciprochlor (Sigma‐Aldrich, USA) for 30 min and quantified at 562 nm using UV–vis spectrophotometer (Perkin Elmer, USA).

### Morphological Analysis of Cells Co‐Cultured with Scaffold Material

To assess the morphology of cells after 3D culture with scaffolds, MC3T3‐E1 and Raw264.7 cells were selected to be inoculated on the surface of porous samples for culture, respectively. After 1 or 3 days of cell‐scaffold co‐culture, cells were lysed with 1% Triton X‐100 (Beyotime, Shanghai, China) followed by Rhodamine‐Phalloidin staining (Invitrogen). Phalloidin and DAPI (Invitrogen) were used to label the cytoskeleton and nuclei. They were then visualized using a CLSM. In addition, co‐cultured cells were fixed with glutaraldehyde, dehydrated with gradient alcohol, and examined using SEM.

### In Vitro Osteogenesis Test

To test the effect of different scaffold materials on osteogenic differentiation of MC3T3‐E1 cells, the cells were inoculated at a density of 1 × 10^5^ cells per well in 24‐well culture plates, co‐cultured with scaffold extract for 24 h and then replaced with osteogenic induction medium (DMEM supplemented with 50 µg mL^−1^ ascorbic acid, 10 mmol L^−1^ *β*‐glycerophosphate disodium, and 10 nmol L^−1^ dexamethasone). The medium was changed every 3 days and cultured in 37 °C, 5% CO_2_ incubator.

### ARS Staining

After 21 days of co‐culture, the samples were rinsed three times with PBS, then fixed in 4% paraformaldehyde for 30 min, and finally the fixed samples were stained with ARS dye for 30 min. The stained samples were rinsed three times with deionized water to remove excess ARS dye. Images of the stained samples were obtained using fluorescence inverted microscopy. For further quantitative analysis of calcium deposition, the stained samples were reacted with 10% cetylpyridinium chloride (Sigma‐Aldrich, USA) for 1 h at room temperature, and the supernatant was taken in a 96‐well plate, and the absorbance at 562 nm was measured by UV–visible spectrophotometer (Perkin Elmer, USA).

### Von Kossa Staining

After 28 days of co‐culture, the samples were washed three times with PBS and immobilized using 4% paraformaldehyde for 30 min, after which the samples were immersed in 5% silver nitrate staining solution and irradiated with UV light for 10 min. After rinsing three times with PBS, images were obtained with an inverted fluorescent microscope.

### Quantitative Real‐Time Fluorescence Polymerase Chain Reaction (qRT‐PCR) Analysis

To assess the promotion of MC3T3‐E1 osteogenic differentiation by bioactive scaffolds, cells were incubated in different scaffold extracts. The expression of specific osteogenic‐related genes alkaline phosphatase (ALP), collagen type I (Col‐1), and runt‐related transcription factor 2 (Runx2) was analyzed by qRT‐PCR assay. After 7 days of culture, MC3T3‐E1 cells were first lysed, and total RNA was extracted and purified from cultured cells using TRIzol Reagent following the steps provided by the manufacturer. After reverse transcription with the HiScript III RT SuperMix kit, the 7500 Real‐Time PCR system (Applied Biosystems, USA) was used to detect the expression of osteogenic genes. Finally, the results were quantified using the 2^−ΔΔCT^ method.

### Immunofluorescence Staining

As described in the previous study,^[^ ^8b^ ^]^ samples were fixed in 4% paraformaldehyde for 15 min after cocultivation with these scaffold extracts in osteogenic‐induced medium for 7 days. Subsequently, after permeabilizing with 0.5% Triton X‐100 for 10 min and infiltrating with 4% BSA at room temperature for 1 h, the samples were stained with primary antibody against OPN (ProteinTech, 22952‐1‐AP, 1:100) or Col ‐1 (Affinity, AF7001, 1:100) overnight at 4 °C, followed by incubation with Goat Anti‐Rabbit IgG (Alexa Fluor647, Abcam, ab150079, 1:200) for 1 h against light after 2–3 times of washing with PBS. Finally, the cytoskeleton and nuclei were stained with phalloidin (red, 561 nm) and DAPI (blue, 405 nm) for 10 min, prior to observation and analysis with CLSM.

### Western Blotting

Cells were placed on ice for 30 min and lysed with immunoprecipitation lysis buffer (Servicebio, G2038) containing phosphatase inhibitors I (MCE, HY‐K0021, USA) and protease inhibitors (MCE, HY‐K0010, USA) for 30 min. Total protein from cell lysates was extracted and a BCA Protein Assay Kit was employed to quantify the protein concentration. After that, 30 µg of protein was sampled for the electrophoresis separation with a 10% sodium dodecyl sulfate (SDS) ‐polyacrylamide gel and then transferred to a polyvinylidene difluoride (PVDF) membrane. After sealing with 5% (w/v) non‐fat milk (Sparks, BD Biosciences, USA) in TBST solution at room temperature for 2 h, the PVDF membranes were incubated in primary antibodies against *β*‐catenin and GAPDH (Proteintech) overnight at 4 °C. At last, the membranes were incubated with corresponding secondary antibodies at room temperature for 1 h, and protein bands were detected using an enhanced chemiluminescence system (Tanon‐5200, Shanghai, Beijing). Image J software was applied to quantify the protein bands density and grayscale values were normalized to GAPDH levels.

### Cell Culture

BMMs were extracted from femurs of 4‐week‐old C57/BL6 female mice according to the previous procedure.^[^ ^30a^ ^]^ BMMs were identified by flow cytometry assay using FITC‐labeled anti‐CD11b. Briefly, BMMs were incubated in *α*‐MEM (10% FBS, 1% P/S) medium and macrophage‐stimulating factor (M‐CSF, 50 ng mL^−1^) at 37 °C in a 5% CO_2_ incubator at rest. The medium was renewed once every 3 days. When cells reached ≈80%–90% fusion, they were digested with 0.25% trypsin for subsequent experiments. All cell handling procedures were performed in a sterile environment.

### TRAP Staining and F‐Actin Ring Fluorescence Staining

TRAP staining was performed according to previous experimental steps,^[^ ^30a^ ^]^ in brief, *α*‐MEM complete medium with scaffold extract solution, 30 ng mL^−1^ M‐CSF and 50 ng mL^−1^ receptor activator of nuclear factor‐*κ*B ligand (RANKL) was added and co‐cultured with BMMs cells for 6 days and stained with TRAP solution for 30 min at 37 °C. Samples were rinsed 2–3 times with PBS and images were collected. F‐actin ring fluorescence staining: cells were fixed with 4% paraformaldehyde for 30 min, then stained with phalloidin ghost pencil ring peptide and DAPI, and finally rinsed with PBS three times. Inverted fluorescence microscopy was used to observe and acquire images. The number of TRAP‐positive cells and F‐actin area were quantified using Image J software.

### Osteoclast‐Related Gene and Protein Expression Tests

Cells were cultured for 3 days and qRT‐PCR was performed as described above to detect mRNA expression of the osteoclast‐associated marker RANK, c‐Fos, and gene expression levels were normalized to the level of the housekeeping gene GAPDH. Immunofluorescence staining was performed as described above, and samples were fixed in 4% paraformaldehyde, then incubated with primary antibody as anti‐c‐Fos or anti‐CTSK at 1:200 dilution and incubated overnight at 4 °C. Finally, the samples were incubated with goat anti‐rabbit IgG for 2 h. Western blotting was performed as described above, using primary antibodies against ERK, JNK and anti‐GAPDH. Protein levels were normalized to GAPDH levels. Image J software was used to analyze the fluorescence intensity, and the protein band grayscale values.

### Experiments on Animals

This experiment was approved by the Animal Ethics Committee of Wuhan University Zhongnan Hospital (ZN2022112), and the experimental method was performed in accordance with the “Guideline for Animal Treatment” (09/30/2006) from the Ministry of Science and Technology of the People's Republic of China. The animal experiments were based on the osteoporosis model and the standard cranial defect model. First, both ovaries were removed, and the osteoporosis model was successfully constructed 3 months after surgery. Scaffolds were implanted in the bone defects. 25 SD rats were randomly and equally divided into five groups of five rats each. 1) CaP group, 2) PDA@CaP group, 3) iPTH+PDA@CaP scaffold (implanted with PDA@CaP scaffold; Intermittent injection of PTH), 4) PTHrP‐1@TCP group, and 5) PP@TCP group. Specimens of skull and femur were obtained after 3 months for testing.

### Histological and Morphological Evaluation

Scanning of the specimen to be measured was performed by micro‐computed tomography (micro‐CT; SkyScan 1276, Germany) and the accompanying Skyscan NRecon, DataViewer and CTAn software for image reconstruction and analysis, including bone volume percentage (bone volume/tissue volume, BV/TV), bone surface area/bone volume (BS/BV), bone mineral density (BMD), bone trabecular number (Tb.N), bone trabecular thickness (Tb.Th), and bone trabecular separation (Tb.Sp). To distinguish between scaffold and bone tissue, the gray value interval was set to ≈100–220 HU for bone tissue and ≈220–255 HU for scaffold. Samples were scanned by micro‐CT and the decalcifications were performed in 10% EDTA at 37 °C for 4 weeks. The tissue was dehydrated, paraffin embedded and sectioned (100–200 µm), and the sections were milled and polished to 40–50 µm. Hematoxylin‐Eosin (HE) staining and Masson's trichrome staining were performed to observe new bone formation, and Goldner's trichrome staining and TRAP staining were performed to assess bone mineralization and bone remodeling. In addition, immunohistochemical staining for ALP, COL‐1*α*1, and RANK was performed to observe the local bone remodeling in the defect area, and immunohistochemical staining for CD206 and INOS was performed to assess the local immune microenvironment. After 3 months of scaffold implantation, organs such as heart, lung, liver, kidney, and spleen of each group were removed, fixed with 4% paraformaldehyde, embedded in paraffin, and sectioned for HE staining to assess the biosafety of scaffolds implanted in vivo for long‐term.

### Statistical Analysis

In this study, all quantitative data results were expressed as mean ± standard deviation (SD), and all experiments were repeated at least three times. Statistical analysis was performed by SPSS 22.0 software (SPSS, Chicago, USA) using one‐way ANOVA and Tukey's test. *p < 0.05, **p < 0.01, ***p < 0.001 were used to determine statistically significant differences.

## Conflict of Interest

The authors declare no conflict of interest.

## Supporting information

Supporting Information

## Data Availability

The data that support the findings of this study are available from the corresponding author upon reasonable request.
