# Supplementary material for: A Recombinant Parathyroid Hormone‐Related Peptide Locally Applied in Osteoporotic Bone Defect
Source: Adv Sci (Weinh). 2023 May 25;10(22):2300516. doi: 10.1002/advs.202300516 (PMC10401080; doi:10.1002/advs.202300516)
Supplement: Supplementary file 1 — Supporting Information [file ADVS-10-2300516-s001.pdf]

ADVANCED SCIENCE
Open Access

Supporting Information

for *Adv. Sci.*, DOI 10.1002/advs.202300516

A Recombinant Parathyroid Hormone-Related Peptide Locally Applied in Osteoporotic Bone Defect

Yi Wang, Yingkun Hu, Shenghui Lan, Zhe Chen, Yufeng Zhang, Xiaodong Guo, Lin Cai and Jingfeng Li**

Supporting Information

A recombinant parathyroid hormone-related peptide locally applied in osteoporotic bone defect

Yi Wang, Yingkun Hu, Shenghui Lan, Zhe Chen, Yufeng Zhang, Xiaodong Guo, Lin Cai*, Jingfen Li*

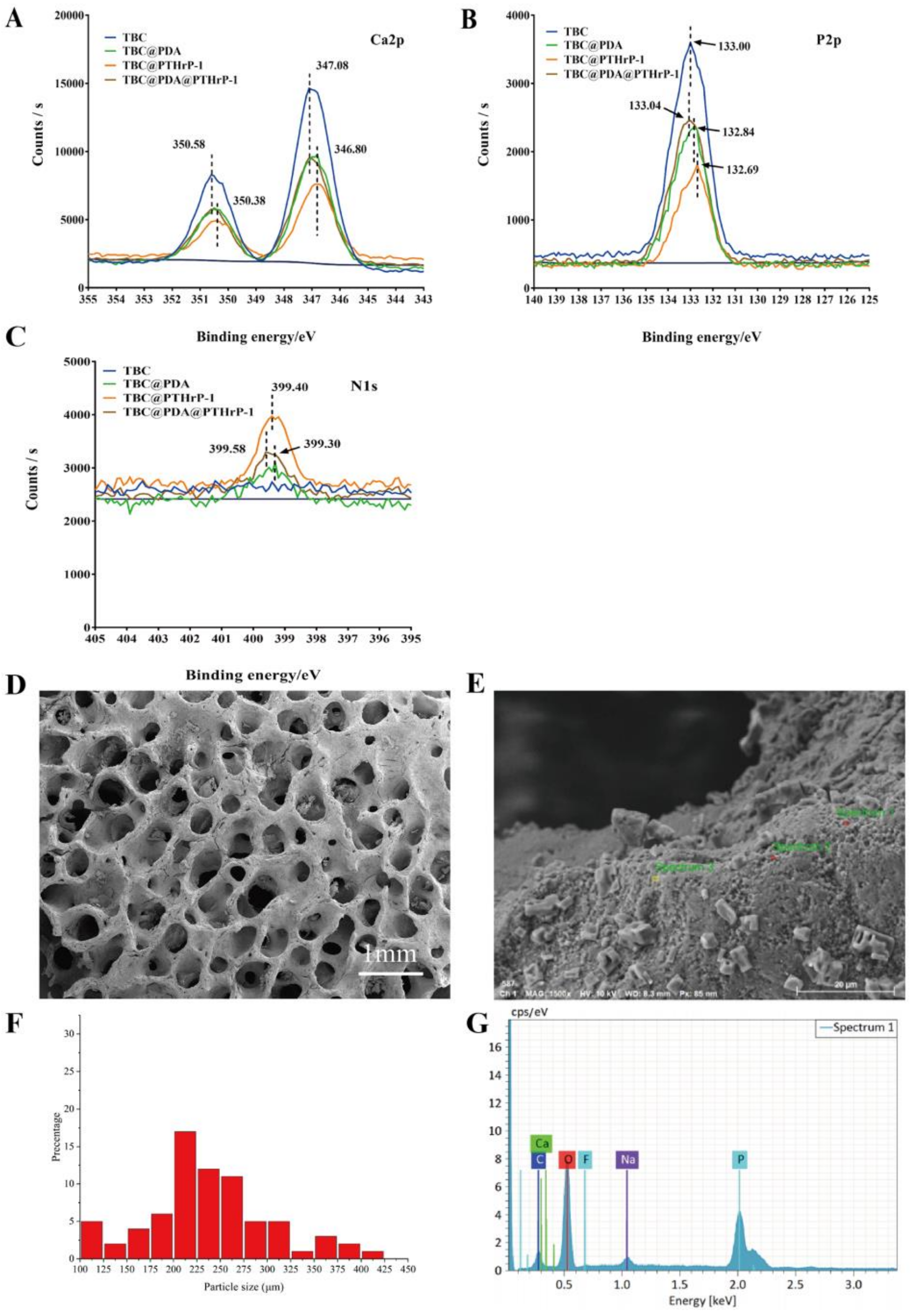

Figure S1 : Point-to-point XPS spectra. The porosity reached 70.20%, which was conducive to the growth of BMSCs, nutrient exchange, and metabolite transport. The pore size distribution of our calcined bone scaffold ranged from 100μm to 425μm , with the most concentrated pore size distribution from 200μm to 225μm (Fig.S1E). In PP@CaP the atom ratio is 1.64 (Fig.3G).

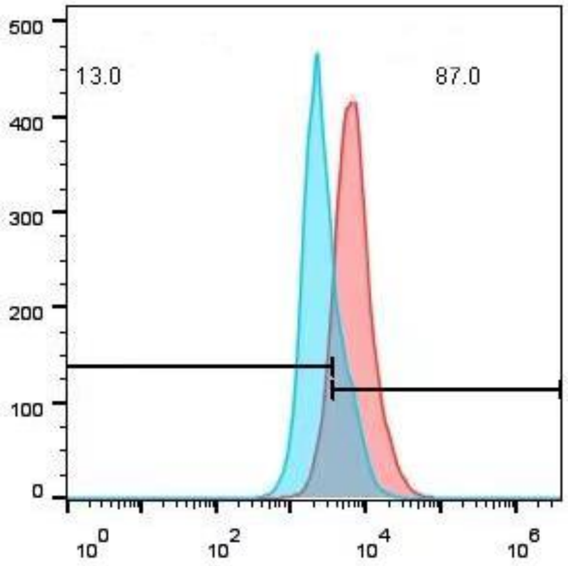

Figure S2: BMMS cells were identified. The percentage of FITC-labeled CD11b-positive cells was 87%.

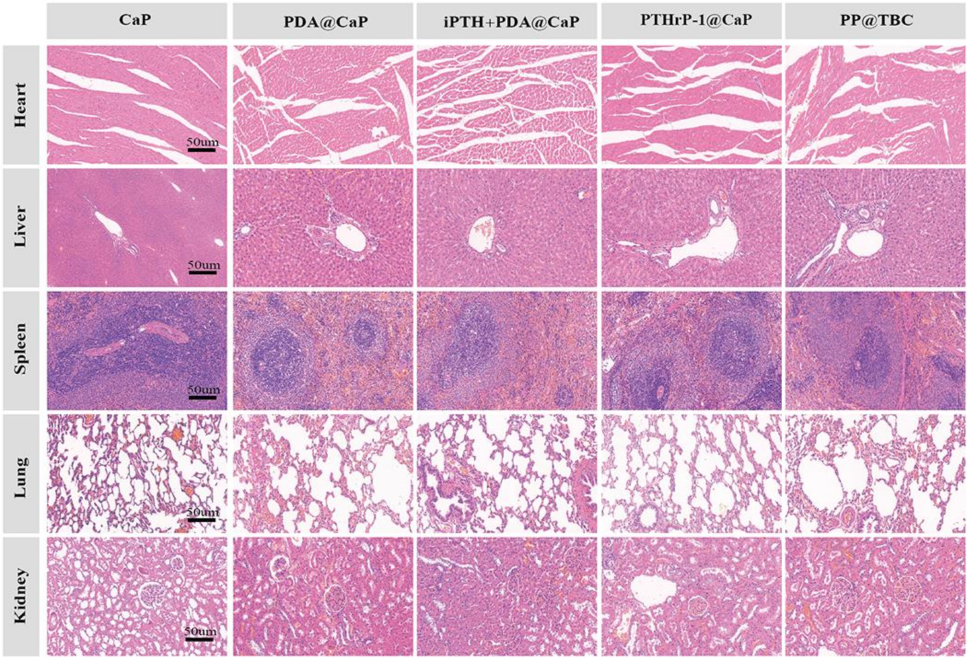

Figure S3: Biosafety of materials, heart, liver, spleen, lung, kidney representative H&E stained images.

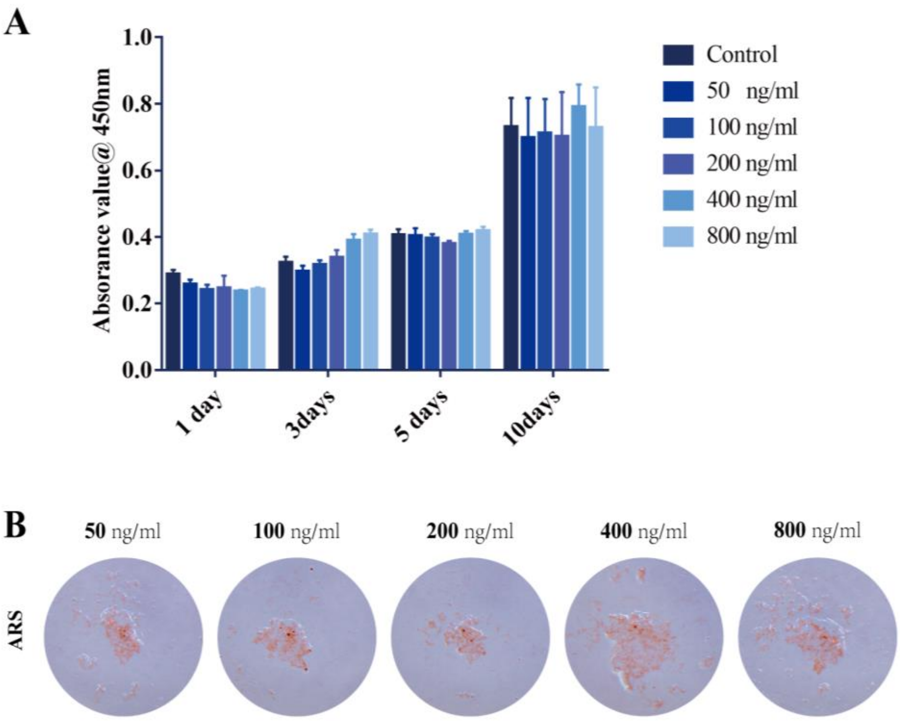

Figure S4: On the 10th day of cell proliferation in the 400 ng/ml group and the 800 ng/ml group, we found that the proliferation effect of the 800 ng/ml group was weaker than that of the 400 ng/ml group with the additive effect of drug concentration (Figure

S4A). Alizarin red staining was performed at 14 days of osteogenic differentiation, and less calcium nodules were formed at 800ng/ml compared with 400ng/ml. Therefore, 400 ng/ml was chosen as the experimental concentration.(Fig. S4B)

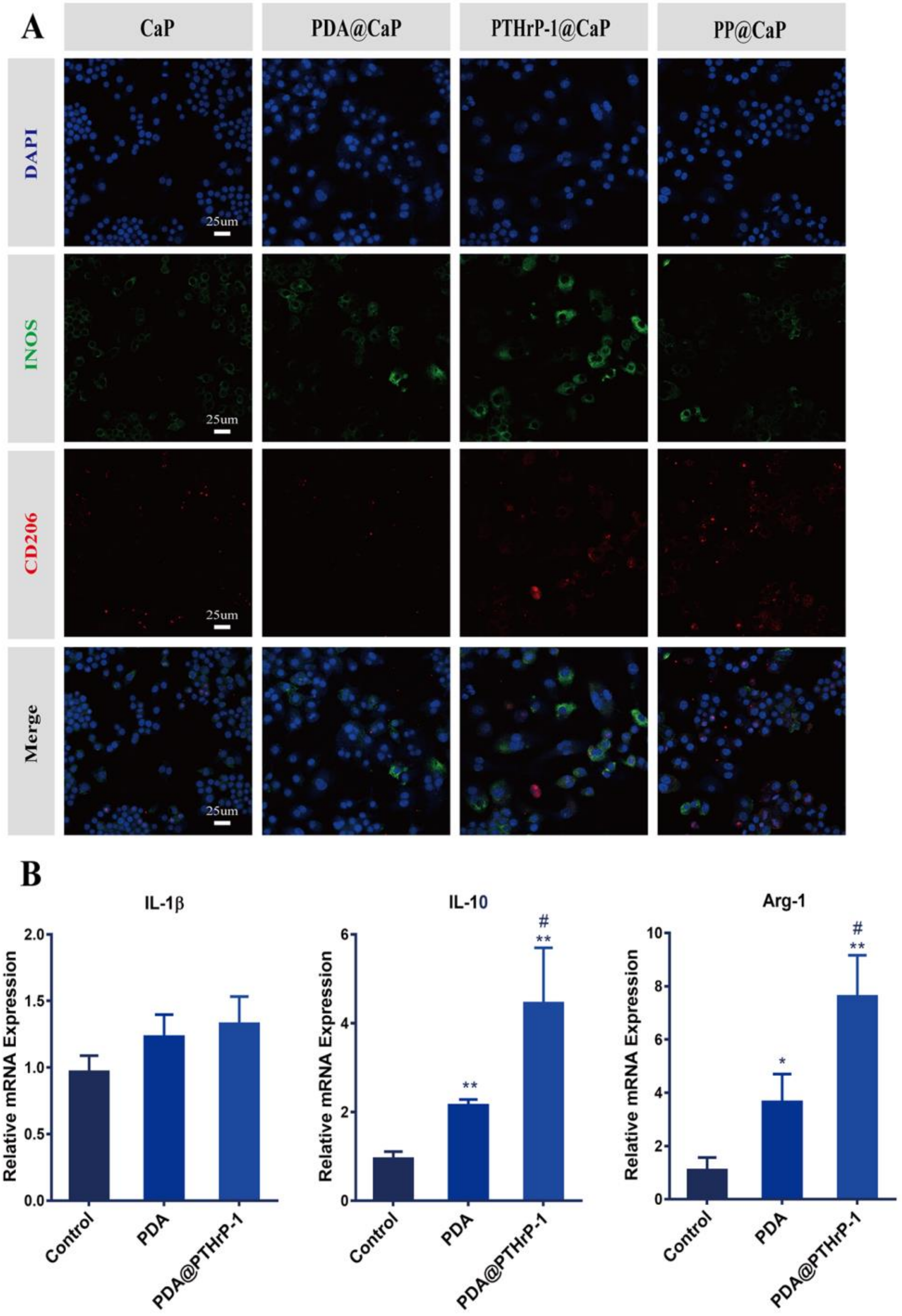

Figure S5: the experimental results showed that the expression level of INOS protein was decreased and that of CD206 protein was increased. Compared with the other groups, the polarization of M1 macrophages gradually transformed to M2

macrophages in the PP@CaP group. In FIG. S5B, the experimental results showed that the IL-1β gene expression levels were slightly increased in the PDA group and the PDA@PTHrP-1 group compared with the control group, but there was no significant difference. The gene expression levels of IL-10 and Arg-1 were significantly increased.Scale bars: 25 μm (A), $^{*}P < 0.05$, $^{**}P < 0.01$ indicate significant differences compared with the control group. $^{\#}P < 0.05$,and $^{\#\#}P < 0.01$ indicate significant differences compared with the PDA@PTHrP-1 group. Data are expressed as mean ± SD (n = 3).
